# Supporting Health Literacy in Youth: An Insight from Academics and Practitioners

**DOI:** 10.3390/healthcare14172725

**Published:** 2026-08-26

**Authors:** Craig Smith, Sarahjane Belton, Hannah R. Goss

**Affiliations:** 1 Department of Public Health and Epidemiology, School of Population Health, Royal College of Surgeons University of Medicine and Health Sciences, D02 HK09 Dublin, Ireland; 2 School of Health and Human Performance, Dublin City University, D09 YH9P Dublin, Ireland

**Keywords:** health literacy, young people, qualitative, health and wellbeing

## Abstract

**Background/Objectives**: Health literacy is a critical determinant of health, closely linked to behaviours, outcomes, and broader social determinants. Given that young people navigate distinct social contexts and health-related challenges compared to adults, understanding how to support their health literacy development during this important period is essential for lifelong wellbeing. This study aimed to explore the perceptions of academics and community-based practitioners regarding the concept of health literacy in young people and the importance of supporting its development. **Methods**: Semi-structured interviews were conducted with 30 participants (academics n = 17, health and wellbeing practitioners n = 9, policy/research n = 4) based across Europe and Australasia, with experience in youth health literacy. Data were analysed using reflexive thematic analysis. **Results**: Three key themes were identified: (i) Building lifelong health agency: From early foundations to transitional autonomy; (ii) Navigating novel health changes; and (iii) Structural inequities and intergenerational barriers in health literacy. **Conclusions**: Participants emphasised that youth health literacy interventions should move beyond didactic, individual-level information delivery toward fostering critical digital competencies and addressing structural barriers through participatory co-design with young people.

## 1. Introduction

Health literacy has been defined as people’s knowledge, motivation, and competencies to access, understand, appraise, and apply health information in order to make judgements and take decisions in everyday life concerning healthcare, disease prevention and health promotion to maintain or improve quality of life during the life course [1]. Health literacy strongly influences health determinants, behaviours, and outcomes across healthcare and health promotion [1,2,3], and evidence shows that establishing health literacy early in life is critical, yet research developing, implementing, and evaluating effective interventions in youth populations remains a priority [4,5].

Health literacy is not a static concept, and it develops over the life course through education, experiences, societal factors, and personal situations [6]. As young people socialise in different environments, contexts, and settings and are exposed to different health-related life situations than adults, they require a different set of health-literacy-related competencies [7]. Given this, and the contextual nature of health needs and health literacy influences [8,9], to effectively operationalise health literacy, it is important to consider the needs of specific populations and contexts (i.e., considering the strengths, needs, and issues faced by young people in today’s world). A holistic understanding of contemporary challenges and practical implementation barriers, from a variety of viewpoints, can support the development of more meaningful interventions [10,11].

This study, as part of a wider project that will also engage with young people directly, aims to gather the perceptions of international academics and community-based practitioners on the concept and importance of health literacy in young people. The aim of this wider project is to work with stakeholders to co-design a digital application that can (a) generate knowledge of young people’s lived experiences of health literacy and (b) be used as an educational tool to support the development of young people’s health literacy. As such, this study represents the initial stages of the project, which seeks to engage stakeholders in evidence gathering as an early stage of the co-design process.

## 2. Materials and Methods

### 2.1. Participants and Recruitment

Ethical approval for this study was granted by the institutional research ethics committee (DCUREC/2023/128). A purposive sampling approach was employed to recruit international academic experts and community-based practitioners in this exploratory, qualitative study. Eligibility for academic participants required a primary research focus on adolescent health, health promotion, or youth health literacy (demonstrated by peer-reviewed publication history within the preceding 5 years). Practitioner eligibility required a minimum of three years of direct, community-based health promotion or educational delivery with youth populations. A total of 85 potential participants were identified through stakeholder mapping, public academic directories, and professional networks and invited via email. Of those, 30 professionals with expertise in youth health literacy (academics n = 17, health and wellbeing practitioners n = 9, policy/research n = 4, based across Europe and Australasia) agreed to take part in this study (see Supplementary Material Table S1 for further details on participants). Sample size adequacy was guided by the concept of information power, considering the study’s specific aim, dense sample specificity, and extensive interview dialogue. It is, however, important to acknowledge a potential voluntary self-selection bias where participants with greater baseline interest in youth advocacy were more likely to respond. All participants were provided with a participant information document before providing informed consent. All participants remained anonymous and were allocated a specific code (e.g., P1). These can be seen in the Section 3 when introducing participant quotes.

### 2.2. Data Collection

A semi-structured interview guide was designed by the research team (see Supplementary Material S2). This was tested and refined through pilot work with a relevant participant, not included within the final dataset, and refinements included clarification of wording, ordering of questions, and the addition of prompts. Within the interviews, the term “young people” was deliberately used as a broad umbrella concept encompassing both children and adolescents to allow participants to speak flexibly across different age groups. Consequently, it should be acknowledged that participants may have had different developmental stages in mind during discussions. One-to-one interviews were conducted by the first author in winter 2023. The majority of interviews (n = 27) were conducted electronically via Zoom video software (Zoom Video Communications, San Jose, CA, USA version 5.17.0), from private locations, and the remaining interviews were conducted in person (in Dublin, Ireland). Zoom has been recommended as an alternative where face-to-face interviews are not possible, to gather rich data. The interviews lasted between 40 and 60 min and were video and audio recorded for subsequent transcription and analysis. No repeat interviews were conducted, no compensation was provided to participants, and transcripts were not returned to participants.

### 2.3. Data Analysis

Reflexive thematic analysis (RTA) [12,13] was conducted by the first author (CS) to analyse the data gathered. The first author (CS) is a male qualitative health researcher with an extensive background in school- and community-based health literacy interventions. RTA is about the researchers’ reflective and thoughtful engagement with their data and their reflective and thoughtful engagement with the analytic process [12]. In this study, the first author approached data collection and analysis with an awareness of his own socio-cultural background and prior assumptions regarding structural health inequalities. While the interview guide was broadly informed by established health literacy concepts, theme generation remained primarily inductive; codes and central organising concepts were derived directly from empirical data rather than mapped onto a pre-existing matrix. An experiential orientation to data interpretation was adopted to explore the participants’ contextually situated experiences and perspectives and was underpinned by a constructionist epistemology. As such, the interpretation of meaningfulness was highly influential in developing codes and themes [14]. Findings therefore reflect an experiential focus on participants’ reported realities with a constructionist critique of the wider socio-cultural and institutional environment.

The data was analysed using Braun and Clarke’s [12,13] six-phase approach to RTA. First, the data was manually transcribed verbatim into Microsoft Word. Each transcript was then read multiple times to ensure familiarity and understanding of the data before systematic coding began. After the full dataset was coded using qualitative analysis software (QSR International’s NVivo 12), the initial themes were generated from the codes. The initial themes were then reviewed, developed, and structured. Throughout this process, the themes were refined, defined, and named. The reporting phase was completed after the codes and themes were developed.

Throughout the data collection and analysis, the lead author maintained a reflexive journal to record key information, interpretations, and insights that were constantly reflected on during the analytical process. This allowed the author to engage with the data, deepening reflexivity. Due to the reflexive and interpretive nature of RTA, the first author predominantly analysed the data. As a method of further interrogating the analysis conducted, the last author (HG) reviewed the reflexive analytical process by sense-checking analysis, exploring alternative interpretations of the data, and challenging analytical assumptions as a critical reflexive dialogue partner in regular meetings throughout the data analysis process [15]. Through iterative reflexive dialogue, the critical friend queried early code labels and probed alternative interpretations, enriching the theoretical depth of generated themes. This approach to analysis encouraged a deepened reflexivity by offering alternative interpretations of the data in a manner that was collaborative and flexible, ultimately leading to richer interpretations of meaning, rather than aiming to achieve a general consensus [15].

## 3. Results

Following the RTA of the data gathered, three themes and subsequent subthemes were generated: (i) Building lifelong health agency: From early foundations to transitional autonomy; (ii) Navigating novel health changes; and (iii) Structural inequities and intergenerational barriers in health literacy (Table 1).

### 3.1. Building Lifelong Health Agency: From Early Foundations to Transitional Autonomy

#### 3.1.1. Supporting Foundational Knowledge and Capabilities That Can Track into Adulthood

Participants emphasised the need to introduce health literacy early, noting that equipping young people with foundational skills at an early age “will stay with them forever” (P7). Participants also highlighted that often health literacy knowledge and skills are introduced too late in life, causing difficulties during transitional periods, such as adolescence and into adulthood. As one participant argued, “we know that we can’t suddenly teach a child at the age of 12 or 13 about healthy lifestyles. It’s too late” (P25). Adopting a gradual, iterative educational process, according to the participants, increases the likelihood that the knowledge and skills will become a part of the individual’s makeup and will stick with them throughout life:

“It’s about developing knowledge and a set of capabilities that continue to develop and redevelop and be refined across the lifespan… if they are practiced continually across childhood and adolescence and across (different) health contexts, then it becomes innate, it becomes part of who you are and your practice as a person”.

(P2)

Within this scaffolded approach, examples of the types of health literacy competencies that should be introduced were also highlighted, with one participant stating that they need to be “taught the tools early to distinguish between bad and good information and to distinguish more than one way to even find the information… then learn how to apply filters of [whether] is it good or bad information…it’s absolutely invaluable for young people” (P21). Extending health literacy beyond functional knowledge of basic facts to critical competencies and application.

Participants also discussed the importance of investing time and resources into supporting the health literacy of younger populations to create an intergenerational impact. By building health agency in youth, public health strategy can disrupt cycles of health disadvantage, shifting the paradigm from costly downstream disease management to upstream structural prevention:

“It is crucial (to support health literacy) in young people because they are the adults of tomorrow, they are the people that we need to invest in now”.

(P21)

The importance of “getting the cycle going” (P14) from an early age “is quite obvious” (P11), and “if we invest the money now in young people to create a healthier next generation of adults, then the disease burden will hopefully be minimised. And the money is well spent” (P21).

#### 3.1.2. Cultivating Youth Self-Efficacy and Autonomy in Developmental Transitions

While the long-term preventive value of health literacy was widely acknowledged, participants equally emphasised its immediate role in fostering self-determination and agency during youth. Health literacy was reframed not as passive compliance with medical advice but as an active mechanism of empowerment that enables young people to interrogate their environments, adapt their daily routines, and make autonomous decisions:

“It shouldn’t just be about information giving and sharing… it should be about being able to empower people to use that information to change their contexts and adapt their circumstances and situations”.

(P27)

This perspective shifts the focus from transactional information sharing to transformative capacity-building. Equipping young people with the “tools at your disposal” (P19) fosters the critical consciousness of reflecting on personal habits, digital consumption, and health trade-offs, asking reflective questions like “why am I spending all this time on my phone?” (P11).

The idea that health literacy empowers young people to exert some control within the health service was also discussed, with one participant stating that:

“Health literacy is not all about them having information, but it’s all about empowerment, and actually giving them a voice in services, which generally are designed to disempower children and young people”.

(P7)

### 3.2. Navigating Novel Health Changes

#### 3.2.1. The Volume of Mis- and Disinformation

Participants highlighted that the sheer volume of mis- and disinformation present in digital content presents a fundamental challenge to young people’s health literacy. The participants stated that a possible strategy to mitigate the risks of consuming such information is to develop the relevant health literacy skills to navigate and manage the masses of information. This was articulated by one participant who stated:

“I think it’s incredibly important (to support health literacy). I think it’s becoming even more important with the over reliance on social media and the amount of content that they’re getting. I think they weren’t getting the same amount of content years ago, and now they’re just getting reams and reams of information constantly spewed at them”.

(P19)

Crucially, participants noted that authority online has shifted away from traditional healthcare professionals toward social media influencers and public figures. The erosion of traditional gatekeeping mechanisms requires young people to constantly evaluate credibility in real time:

“A lot of the information they’re getting would be online, on social media and Tik Tok these days… so good health literacy would be being able to recognise that just because an influencer or a sports person says something… it doesn’t necessarily make them an expert”.

(P15)

#### 3.2.2. The Digital Sphere

Along with the risks associated with the volume of mis- and disinformation young people are exposed to online, the participants were also concerned with the high level of screen time youths are consuming in the “digital sphere” (P15), as well as the addictive nature of the prominent online platforms (and the health concerns that may arise as a result). Participants described an evolving media landscape in which traditional health education curricula struggle to keep pace with the speed, volume, and sophistication of online health information and novel health risks. Rather than dealing with static health messaging, youth are embedded in dynamic digital environments where social media architectures actively shape health perceptions, normative behaviours, and consumption choices. One participant said:

“I think the online space (is dangerous for young people). It’s so funny because that used to be one thing and now it’s everything. It’s not even just about the (mis- and dis-) information. It’s about how people are choosing to spend their time”.

(P20)

Participants further described how they believed platform features exploit neurobiological mechanisms, creating continuous cycles of engagement “just scrolling and scrolling (through social media)”, and the “dopamine hits” associated are harmfully “wiring the brain” (P11). Participants noted how spending large quantities of time online is having a knock-on effect on other health behaviours, such as sleep. One participant discussed how young people would rather spend time communicating with friends late at night rather than going to sleep at a reasonable hour:

“They (the young people) know that they should be getting more sleep than they get, but what’s more important to them is making sure that they’re not missing out on a conversation with their friends at 11 o’clock at night… it’s the culture these days”.

(P3)

Although participants were concerned, they were also keen to point out that a considered approach is needed when tackling this issue and that it is important not to “demonise” the use of social media and other online platforms. It was highlighted that “developers have designed it (social media platforms) in a way that’s extremely addictive for you (the users)” and that “it is not them (the young people) that are problematic” (P11).

Participants went on to mention that the online world “can be a tool for good when used in the right way” and that “it is amazing what resources are on there (online)… so just to cast all that stuff as dark, ominous force of evil is wrong” (P7). Instead of demonising, participants suggested that there is a need to “work with kids” to “equip them (the young people) with some critical thought about what makes that (social media usage) dodgy. What makes that questionable. Why looking at lots of that would influence them…so let’s not treat kids stupidly, because they’ll look (online) anyway” (P7).

### 3.3. The Rapid Emergence and Impact of Vaping

Participants also discussed how vaping in particular is a novel challenge that has become a considerable health challenge for young people in recent times:

“You can almost see the vaping story following the exact same path as the smoking story from the scientific research and the health implications… so it’s definitely vaping (that is the greatest health concern)”.

(P3)

The rapid rise of vaping among youth serves as a clear exemplar of how novel, commercially driven health risks can outpace static health educational curricula. Participants discussed how urgent action is needed to avert the risks of vaping, with many noting that supporting health literacy in young people could have a positive impact in tackling the misinformation around vaping and reducing its prevalence. However, despite the participants’ awareness of some educational and control strategies within schools, these had limited impact to date. As one participant, a teacher, noted:

“We are really cracking down on vaping (in schools), and we’ve got posters all over the school, and we’ve got vape detectors, and we’ve tried everything. And they (the young people) know vapes aren’t good for you, and they know where to find (health) information. And we’ve (the teachers have) told them, but the decision that they make, I don’t think that they perceive the health risks to be greater than the social risks of not vaping”.

(P4)

Participant insights suggest that combating such rapid health risks requires moving beyond static facts, equipping youth instead with critical health literacy to unpack information, peer exposure, and commercial influence in real time, impacting vaping behaviour and acting as a preventative measure against future health trends.

### 3.4. Structural Inequities and Intergenerational Barriers in Health Literacy

When discussing the importance of health literacy in young people, participants highlighted that systemic inequities deeply influence health capabilities and engagement across specific sociodemographic groups. Rather than viewing variations in health behaviours as individual failures or personal choices, participant accounts underscored how institutional design, resource distribution, and socio-economic realities shape and constrain health agency.

#### 3.4.1. Deprivation and Socioeconomic Constraints

It was highlighted that place-based conditions heavily structure health opportunities, emphasising that “the geography of where they (young people) live” (P10) plays a major role in their health literacy and health choices. Living in socioeconomically marginalised communities exposes young people to environments with limited access to health-promoting resources and pervasive environmental stressors. As one participant observed, “90% of young people in these areas are at risk of going down the wrong path because of the environment that they live in. There’s so much temptation” (P24).

Furthermore, participants also described how health systems and educational messaging often operate on assumptions of autonomy, failing to accommodate the lived reality of structural poverty. Where survival needs are paramount, immediate material concerns take precedence over preventative health behaviours:

“So, if you’ve got priorities in life, so if you’re living in poverty or unemployment, or you’re living in a different… difficult family situation, health isn’t (a priority). I guess, those of us in the health field think health is really important, but for a lot of people, it isn’t… getting what I am going to eat tonight is more important”.

(P8)

Traditional public health interventions were heavily criticised for taking a “very middle-class” (P1) approach that assumes agency where choice is structurally constrained. Participants observed that institutional approaches often rely on normative standards that inadvertently lead to systemic shaming rather than support:

“…We’re being very judgmental, and we’re shaming kids who really have enough going on in their lives already. They don’t need to be hearing that. So, if kids aren’t eating well, or they’re not exercising, there is (a belief that there is) some degree of choice in it. But sometimes it’s down to not having choice”.

(P1)

Beyond environmental constraints, participants pointed to structural flaws within the delivery of health services themselves. Rather than adapting services to meet the needs of historically marginalised or underserved communities, systems often marginalise groups that require tailored support. It was also mentioned that “there are lots of populations that as health services and health professionals, we don’t try hard enough to work with” and that often it is a case of “working with the ones that want to engage”. Thus, the participants felt that vulnerable populations were “at risk of being excluded or being seen as not worthy of being empowered” because there is often an attitude that “they’re a bit hard (to work with), aren’t they?” (P7). Resulting in a tendency within institutional practices to prioritise engagement with populations that fit standard service models.

#### 3.4.2. Intergenerational Transmission of Health Disadvantages and Educational Gaps

The influence of family background on youth health literacy was framed not as an inherent failure of parenting but as the intergenerational transmission of structural advantage or disadvantage. Participants observed that young people’s health decisions are continuously conditioned by the resources, health literacy capital, and social context of their broader environment, demonstrating how historical and structural inequities compound across generations. Participants noted that “you are influenced by the people around you, so if your parents did not have them (health literacy) skills, they don’t know how to teach you them [those] skills” (P24) and that “you model the behaviour you see, especially when you are a child or young person” (P26).

Other participants noted that parents can be particularly difficult to engage when it comes to youth health interventions due to their busy schedules and various demands. Therefore, participants highlighted that by supporting the health literacy competencies and knowledge early in life, the challenge of attempting to engage parents may be reduced:

“Parents are notoriously difficult to engage, being busy…. there’s so much going on… So, the adolescents that we’re working with now, hopefully, they’ll go on and some of it will stick for their future children”.

(P6)

#### 3.4.3. Marginalisation and Cultural Mismatch in Standardised Health Communication

Beyond economic disparities, participants emphasised that institutional health and educational systems are predominantly structured around normative, dominant-culture frameworks. This structural alignment actively marginalises minoritised communities—in this study, Traveller populations, gender-diverse youth, and migrant or refugee families were explicitly mentioned—by failing to account for their distinct social contexts and life conditions. These populations were described as “vulnerable” (P10) or “disadvantaged communities” (P27) by the participants. Participants highlighted the travelling community as a population at risk of poor health outcomes due to their “lifestyles being very different, in terms of education. They may not engage with schools as much” (P26). Furthermore, participants mentioned that “their environments, their housing, their risk of poverty” (P27) also put them at a disadvantage. One participant noted that “the suicide rates in men are six times greater than men in non-travelling community populations” (P26).

Participants identified non-binary and gender-diverse youth as another group systematically underserved by existing health infrastructure. Institutional health communication remains heavily reliant on binary assumptions, creating information vacuums and hostile care environments for those outside these norms. One participant said, “I think that those people that don’t categorize into that binary boy or girl are at potential risk” (P12). They went on to explain that “there is just not that much information out there for them” and that:

“If you don’t fall into that (binary) category, there is the discrimination and stereotyping and all of that that goes with it. So, if you feel like you don’t fit into a category and you don’t know where to go to support your health, whether it’s mental health or whatever it might be, you could leave something untreated, and it can get worse”.

(P12)

This absence of inclusive, tailored health information represents a structural failure to provide adequate resources. The participant emphasised that this is an institutional issue, paired with pervasive social stigma, creating a major barrier to accessing timely care.

Finally, participants discussed how healthcare and social services frequently fail to accommodate the complex structural realities of “asylum seekers, refugees and immigrants” (P7). Rather than viewing linguistic barriers or caregiving structures through a deficit lens, participant accounts suggest how rigid health systems fail to adapt to diverse household compositions and socioeconomic pressures. Language access remains a primary structural barrier, with participants pointing out that “apart from anything else, language can be a difficulty” (P27) when navigating standard health systems. Moreover, institutional interventions often fail to account for the division of labor and household responsibilities common in newly arrived or socioeconomically constrained migrant families:

“The children might be one of eight kids or one of nine kids. And unfortunately, it does make it difficult because when they get home, their parents are at work. So, as an older sister, for example, they then have to cook dinner for their family. Or when they get home, they have to look after their siblings. So, it’s really hard to get those students to see how they can make health decisions when they have different situations to deal with at home. Again, it comes down to culture”.

(P26)

Ultimately, participant narratives suggest that when health communication relies on a one-size-fits-all model, it inherently privileges dominant socio-cultural groups while penalising minoritised populations for navigating unique, structurally imposed realities.

## 4. Discussion

Synthesised insights from differing perspectives (academics and practitioners) propose that rather than treating health literacy as a passive, individual-level information processing task, youth health literacy operates as a dynamic, context-sensitive capability heavily constrained by social, commercial, and structural environments. Supporting youth health literacy requires moving beyond traditional information provision toward building critical, context-sensitive capabilities across the life course.

Study participants highlighted specific contemporary health challenges that young people face, such as vaping, misinformation, and time spent in the digital sphere. While prior literature widely documents youth exposures to vaping and social media and the negative implications of these emerging health challenges [16,17,18,19,20], our findings highlight potential underlying mechanisms driving these behaviours and position health literacy as a way to combat these new and future emerging health trends. Along with the well-known influencers of young people’s health behaviours, such as peers, parents, and the school environment [21], there are many newly emerging social and environmental factors impacting healthy choices [17]. It is well known that unhealthy products are often marketed at young people, which has been evidenced by the tobacco industry for decades [22]. Typical forms of contemporary marketing include paid placements of products, sponsorship of sporting events and concerts, and colourful packaging [23]. As young people are high consumers of entertainment media and are more susceptible to media influence due to their growing focus on personal image and identity [17], marketing companies can take advantage of their likelihood to identify with what a public figure is advertising [23]. In modern digital ecosystems, health literacy extends far beyond evaluating basic facts; it requires navigating sophisticated commercial determinants of health. As expressed by participants in this study, young people frequently understand the long-term health risks of vaping or late-night screen use, yet choose to engage in them because immediate social risks (e.g., peer exclusion, missing late-night digital connection) outweigh perceived health consequences. These insights are vital to consider in relation to traditional health education, which often relies on risk communication and information provision and therefore views health decisions as purely rational, individual choices divorced from social context. Within research, understanding of health literacy must continue to incorporate critical digital and commercial health literacy [5]. This extended theoretical framing views youth not merely as passive recipients of information but as active digital citizens navigating algorithmically structured environments and profit-driven health claims. Furthermore, public health policy should follow this view and transition from more traditional individual-blame paradigms toward regulating structural environments. It should also be considered that targeting critical health literacy, as opposed to explicit health topics, which vary in importance over time and context [5,24,25], may be a more sustainable and impactful approach to health education.

Contrary to deficit-based models that attribute poor health decisions to low personal or parental literacy, our findings frame sociodemographic disparities as products of institutional mismatch and structural inequality. Wider literature acknowledges that demographic and social factors, such as home address, education attainment, and social support, have been closely linked with levels of health literacy [9,26,27]. Participants within this study emphasised that many health promotion strategies reflect a middle-class ethos that implicitly judges or shames youth facing severe socio-economic disadvantage. Rather than viewing low parental health literacy as a deficit, participants suggested that early, community-embedded youth health literacy initiatives could potentially serve to disrupt intergenerational health disadvantages. To do this effectively and sustainably, health educators and community practitioners must move away from didactic, top-down instruction and work ‘with’ rather than ‘on’ participants [28]. Adopting a participatory approach, which involves the relevant stakeholders throughout the process, is believed to lead to initiatives based on contextual factors, which increases the likelihood of intervention relevance, participant buy-in, subsequent better outcomes and reduced research wastage [29,30]. As such, although this study has provided valuable insights into the importance of supporting health literacy in young people, as perceived by international academics and community-based practitioners, there is still, however, a need to gain an understanding from the perspectives of the young people themselves. Thus, the authors recommend that a critical next step in attempting to support the health and health literacy of young people is to engage with this target age group, across different contexts, to first explore the health literacy strengths and needs and subsequently develop methods to overcome context-specific health challenges.

### Limitations

Several methodological limitations must be considered when interpreting these findings. First, the sample was subject to self-selection bias, as the 30 participants who agreed to participate out of 85 contacted may hold heightened professional interest in youth advocacy. Second, while the study captured rich cross-sectoral perspectives, participants were predominantly based in high-income Western countries, limiting the direct transferability of findings to low- and middle-income health system contexts. In addition, data collection relied predominantly on online video interviews (n = 27) compared to in-person sessions (n = 3), which, while expanding geographic reach, may have subtly influenced interpersonal rapport, non-verbal communication, and overall depth of qualitative dialogue. Third, data collection relied on adult expert and practitioner perspectives as proxies for youth experiences. As a single, standardised age definition was not imposed during interviews, participants’ insights reflect varying developmental stages across childhood and adolescence. This represents an interpretive limitation, as health literacy needs, autonomy, parental involvement, and digital interactions vary significantly across developmental phases. While these professional insights offer valuable systemic overviews, they do not replace direct empirical research capturing the lived experiences and agency of young people themselves. Finally, as a qualitative inquiry rooted in reflexive thematic analysis, interpretations are inherently mediated by the research team’s positionality and epistemological stance.

## 5. Conclusions

By integrating perspectives from both international academics and community practitioners, this study provides a unique, cross-sectoral approach and qualitative insight into youth health literacy. The findings suggest that supporting health literacy early in life is not merely an exercise in functional health instruction but a potential modifiable mechanism for fostering lifelong health agency, structural equity, and intergenerational wellbeing. Modern health challenges, such as the proliferation of misinformation, the pervasiveness of the digital environment, and the growing prevalence of vaping, pose significant risks to youth health. Rather than focusing on a narrow, individualised view of health behaviours, these challenges reinforce the need to promote critical thinking and digital health literacy skills.

Furthermore, the study draws attention to the social, economic, and structural inequities that influence health literacy. Addressing health disparities requires moving away from deficit-based individual paradigms and normative assumptions. Instead, public health systems must address institutional mismatch, language barriers, and material constraints while adapting services to support marginalised, minoritised, and socioeconomically underserved youth. However, meaningful progress requires inclusive, participatory approaches that engage young people as co-creators of health literacy initiatives, ensuring interventions are relevant, context-sensitive, and empowering.

In summary, supporting youth health literacy is a public health imperative that demands structural reform and co-designed innovation. Future research should prioritise the voices of young people to ensure strategies not only reflect their lived experiences but also harness their potential as active agents in their own health journeys.

## Figures and Tables

**Table 1:** Perceptions of international academics and community-based practitioners on the concept and importance of health literacy in young people.

Theme	Central Organising Concept	Subtheme
Building lifelong health agency: From early foundations to transitional autonomy	Developing foundational health literacy capabilities across childhood and adolescence to foster self-efficacy, agency, and active decision-making	Supporting foundational knowledge and capabilities that can track into adulthood
		Cultivating youth self-efficacy and autonomy in developmental transitions
Navigating novel health changes	Critical navigation of evolving digital environments, commercial influences, online misinformation, and emerging risks	The volume of mis- and disinformation
		The digital sphere
		The rapid emergence and impact of vaping
Structural inequities and intergenerational barriers in health literacy	Recognising how material deprivation, institutional design, intergenerational health disparities, and cultural or linguistic mismatches shape health literacy options	Deprivation and socioeconomic constraints
		Intergenerational transmission of health disadvantages and educational gaps
		Marginalisation and cultural mismatch in standardised health communication

## Data Availability

The data presented in this study are available on request from the corresponding author due to privacy restrictions.

## Supplementary Materials

The following supporting information can be downloaded at https://www.mdpi.com/article/10.3390/healthcare14172725/s1, Table S1: Participant characteristics; File S2: Interview guide.
