# Supplementary material for: Supporting Health Literacy in Youth: An Insight from Academics and Practitioners
Source: Healthcare (Basel). 2026 Aug 26;14(17):2725. doi: 10.3390/healthcare14172725 (PMC13564714; doi:10.3390/healthcare14172725)
Supplement: Supplementary file 1 [file healthcare-14-02725-s001.zip › File S2- Interview guide-edited.pdf]

S2. Interview guide.

1. What are the first things that you think of when you hear “health literacy”?
2. Can you tell me about your understanding of health literacy?
3. How important do you think health literacy is for young people?
4. How can we support the development of HL?
5. What do you feel are the biggest challenges facing the development of HL?
6. What’s needed to overcome these challenges?
