# Supplementary material for: Supporting Health Literacy in Youth: An Insight from Academics and Practitioners
Source: Healthcare (Basel). 2026 Aug 26;14(17):2725. doi: 10.3390/healthcare14172725 (PMC13564714; doi:10.3390/healthcare14172725)
Supplement: Supplementary file 1 [file healthcare-14-02725-s001.zip › Table S1. Participants Characteristics (20-8-26)-edited.pdf]

Table S1. Participant characteristics.

Participant	Country	Occupation
1.	Ireland	Academia
2.	Ireland	Academia
3.	Ireland	Health and wellbeing practitioner
4.	Ireland	Academia
5.	Australia	Health and wellbeing practitioner
6.	England	Health and wellbeing practitioner
7.	England	Health and wellbeing practitioner
8.	New Zealand	Academia
9.	South Africa	Academia
10.	South Africa	Academia
11.	England	Academia
12.	Ireland	Health and wellbeing practitioner
13.	Ireland	Health and wellbeing practitioner
14.	Ireland	Health and wellbeing practitioner
15.	England	Academia
16.	Northern Ireland	Researcher
17.	Northern Ireland	Policy and research
18.	Australia	Academia
19.	Australia	Academia
20.	Australia	Academia
21.	England	Academia
22.	Ireland	Policy
23.	Ireland	Academia
24.	Ireland	Policy
25.	Portugal	Academia
26.	England	Academia
27.	Netherlands	Academia
28.	Germany	Academia
29.	Ireland	Health and Wellbeing practitioner
30.	Ireland	Health and Wellbeing practitioner
